# Successful management of bulky osseous metastasis of renal cell carcinoma with selective arterial embolization and radiofrequency ablation: a case report

**DOI:** 10.4076/1757-1626-2-6484

**Published:** 2009-06-29

**Authors:** Christos Emmanouilides, Danai Chourmouzi, Ioannis Dedes, Dimitrios Pantoleon, Polikseni Mantziari, Antonios Drevelengas

**Affiliations:** 1 Department of Medical Oncology, Interbalkan Hospital10 Asklipiou st, 57001 Pylaia, ThessalonikiGreece; 2 Department of Diagnostic Radiology, Interbalkan Hospital10 Asklipiou st, 57001 Pylaia, ThessalonikiGreece

## Abstract

The case of a 64-year-old male patient who presented with a bulky osseous tumor metastasis from renal cell carcinoma is reported. The metastatic lesion extended from the acetabulum of the left iliac bone into the iliosacral joint.

Treatment plan included selective arterial embolization followed by transdermal radiofrequency ablation, followed by consolidation irradiation. This sequence resulted in considerable necrosis of the bulk of the tumor and creation of a large tissue deficit, which healed over the period of several months despite anti-angiogenetic systematic treatment. The patient remains in good state of health, 20 months after the procedure. This case illustrates the usefulness of the embolization-ablation sequence in controlling large osseus metastasis.

## Introduction

Renal cancer is a relatively uncommon malignancy which is however diagnosed with increasing frequency [1]. It presents several challenges to the treating physician because of its resistance to conventional anticancer treatments and its relative sensitivity to immunological or targeted therapies. Metastatic renal cell carcinoma has been an area of important medical advances recently, as it has been shown that it is responsive to antiangiogenic therapy. As a result of the improved outcomes thus achieved, the median survival of patients with metastatic disease is reported to approach 2 years in recent trials. Nevertheless, patients may experience significant morbidity and overall impairment of their quality of life. Bone is a common site of metastasis which is often symptomatic and debilitating. Besides systematic treatments, the management of bone metastasis may include localized modalities, more commonly external beam irradiation. The success of this method is often limited as renal cell carcinoma is known to be relatively radioresistant [2].

Alternative approaches in the management of renal cell carcinoma include local embolization [3-5] or ablation methods. Embolization involves catheterization of the feeding artery of the tumor and embolization with or without instillation of anticancer agents. Embolization has been used to treat primary renal cell carcinoma when resection is not possible, with considerable success. It has occasionally been used to treat metastatic disease as well. Radiofrequency ablation [6] is another localized approach whereby thermal tumor necrosis is achieved, usually via transdermal route. Limitations include large tumor size, accessibility of the tumor and sensitivity of adjacent structures.

The combination of embolization and radiofrequency ablation has not been adequately studied in the metastatic setting [7]. It offers the advantage of ablating an already injured and hypoxic cancerous mass, therefore enhancing perhaps the anticancer effect. We hereby report a case of a patient with a bulky osseous tumor metastasis from renal cell carcinoma, successfully managed with a pre-planned sequence of embolization followed by transdermal radiofrequency ablation.

## Case presentation

**Figure 1.**
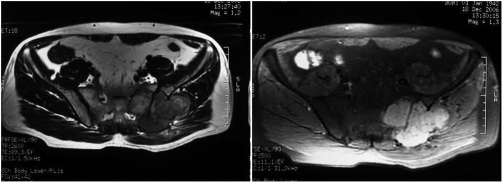
Pre-therapy CT images. **(A)** Axial T2-weighed image of the pelvis: shows a large lobulated mass with intermediate signal intensity. **(B)** Post contrast T1W Fat Sat image: shows intense enhancement of the mass.

A 64-year-old Greek male patient underwent a radical nephrectomy for a stage 2, grade 3, renal cell carcinoma in September 2001. Five years later, he presented with left hip pain and he was eventually diagnosed with biopsy proven relapsed disease. Imaging with CT scans and bone scan revealed a prominent bulky metastatic lesion extending from the acetabulum of the left iliac bone into the iliosacral joint, a metastatic lesion of the second left rib, as well as several metastatic pulmonary nodules (Figures 1a,b). He was started on anti-angiogenetic TKI agent sorafenib and biphosphonates.

**Figure 2.**
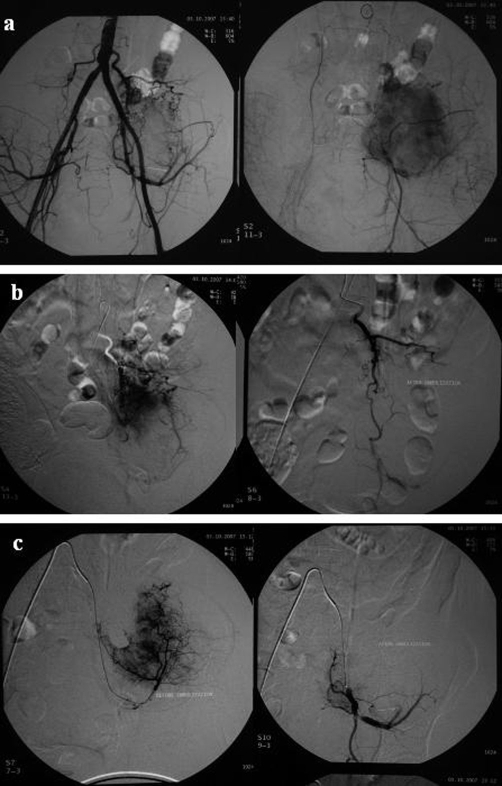
**(A)** Anteroposterior view of aortogram demonstrates a prominent tumoral blush. The feeding arteries of the lesion originated from both 4th lumbar and left internal iliac artery (arrows). **(B)** Selective catheterization of lumbar artery (left) and left internal iliac artery (right). **(C)** Follow-up angiography after embolization shows no residual tumoral blush.

**Figure 3.**
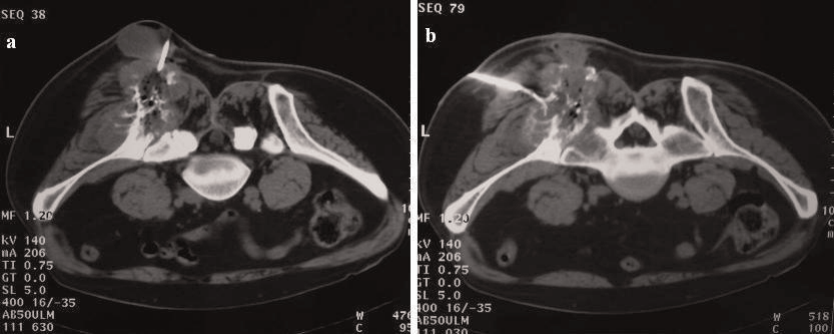
Unenhanced CT scans obtained during ablation show the large exophytic ileac mass **(A, B)**. The radio frequency needle inserted into the mass under CT guidance through posterior approach.

**Figure 4.**
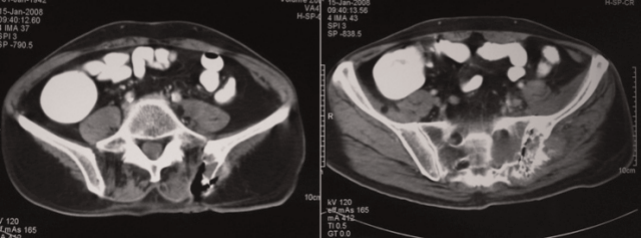
Follow-up CT scan images 3 months post procedure show a large tissue deficit corresponded to ablated tissue. Note the air within cavitary lesion.

**Figure 5.**
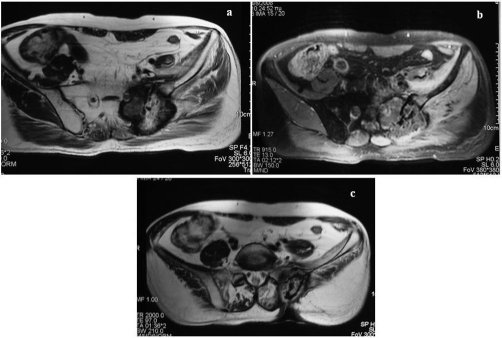
Post therapy MR follow up images **(A, B, C)** 10 months post procedure show shrinkage of the mass and the cavitation as well and hypointense area corresponded to ablated tissue.

After a year of treatment, in October 2007, a necrotic ulceration appeared on the left flank area, proven by CT to be an extension of the increasing metastatic left iliac focus, which measured 8 cm in maximum diameter, associated with increasing pain. The lesion was considered too large to be controlled with irradiation. The patient underwent an arterial embolization of the metastasis using a femoral artery approach (Figure 2). One week later, the same mass was treated by radiofrequency ablation (RFA) (Figure 3). The procedure was well tolerated overall and led to significant pain reduction (Figure 4). However, the ulceration rapidly enlarged and evolved into a crater-like deficit of the skin and subcutaneous tissue 5 cm deep and of 5 cm diameter (Figure 5a). It was decided to be managed conservatively with frequent local wound care. Antiangiogenic treatment was suspended for a period of a month to allow for wound healing. However, during this time the pain recurred and a follow up CT showed evidence of extension of the large lesion medially towards the spinal canal; the bulk of the lesion was necrotic and a large tissue deficit was documented. The patient received external beam irradiation with symptomatic control (4750 cGy in 19 sessions) followed by initiation of sunitinib and biphosphonates. The patient continued improving, regained ambulation, and his disease elsewhere remained stable for over 16 months. His necrotic ulcer has shrunk considerably to less than 0.5 cm in diameter (Figure 5b,c). Because of intolerance to sunitinib, after 4 months of treatment, he was switched to bevacizumab. The patient currently, as of October 2008, remains in good state of health, with slowly progressing pulmonary nodes.

## Discussion

Bulky bony metastasis of the axial skeleton of a relative radioresistant tumor such as renal cell carcinoma is usually a problematic medical situation associated with significant morbidity. In the case of our patient the comorbidity was exacerbated by the presence of skin infiltration and ulceration caused by direct contiguous tumor invasion. A conventional approach would have been to treat with external beam irradiation and analgesics. Such an approach would lead to local failure soon, given the tumor bulk. The sequence of embolization followed by radiofrequency ablation caused extensive necrosis of the tumor bulk and creation of a large tissue deficit, which was at first considered of ominous nature, but as it appears heralded a favourable outcome. The significant tumor reduction thus achieved may have resulted in the increased efficacy of the subsequent therapies provided, namely external beam irradiation and further antiangiogenetic therapy. Of note, excellent wound healing with secondary intent proceeded over the subsequent year, without complications and in spite of antiangiogenetic therapy. It is conceivable however that the administration of subsequent therapy may have delayed the healing process, although without apparent untoward effects.

Therapeutic embolization of osseous metastases has been previously reported in small number of patients with a variety of tumors [3,8]. This procedure has even been reported to result in a rapid resolution of neurological symptoms if they were present [4]. In a report of five patients with renal cell carcinoma the majority of patients experienced significant relief of pain and improvement of their overall clinical condition which lasted for several months [5]. In the case of our patient, it was believed that selective arterial embolization might have been inadequate to control the bulky tumor mass, not only because of its size, but also because of its resistance to pharmaceutical antiangiogenic manipulations. Therefore, the treatment plan included immediate post-embolization radiofrequency ablation (RFA). This technique is usually performed in patients with soft tissue metastases, mainly of the liver. In renal cell cancer RFA has been applied as an alternative to surgery for the definitive treatment of primary tumors [9]. For the purpose of treating painful osseous metastases it has occasionally been used in combination with osteoplasty, again with considerable success [9,10]. To our knowledge, the combination of arterial embolization followed by RFA has only been reported in two cases of non metastatic renal cell carcinoma for the treatment of the primary tumor when resection was not possible [7,9]. Despite the immediate beneficial result, the location of the mass in the proximity of the spinal canal raised the concern of possible under-treatment of the medial part of the tumor. For that reason, external beam irradiation was added, at a point when the patient complained of increasing pain, without clear radiographic evidence of tumor progression. It is noteworthy that our complex therapeutic intervention resulted in the creation of a very large tumor deficit, which did not interfere with the patient’s quality of life and gradually healed with secondary intent.

In conclusion, our case illustrates the usefulness of the sequence of localized treatment for the management of bulky osseous metastasis. Such a specialized treatment approach should be entertained in selected patients as it can yield long lasting favourable outcomes.

## Abbreviations

CT: Computed tomography
RFA: Radiofrequency ablation
